# The Value of Glycemic Gap for Predicting Mortality in ICU in Patients With and Without Diabetes

**DOI:** 10.1155/jdr/4563928

**Published:** 2025-02-14

**Authors:** Ran Lou, Li Jiang, Meiping Wang, Tingting Wang, Quan Si, Weixue Su, Nan Wang, Yuyan Liu, Ting Chen, Qi Jiang, Bo Zhu

**Affiliations:** ^1^Department of Critical Care Medicine, Xuanwu Hospital Capital Medical University, Beijing, China; ^2^Department of Epidemiology and Health Statistics, School of Public Health, Capital Medical University, Beijing, China; ^3^Department of Critical Care Medicine, Fu Xing Hospital, Capital Medical University, Beijing, China

**Keywords:** ADAG, critically ill patients, glycemic GAP, hypoglycemia, stress-induced hyperglycemia

## Abstract

**Objective:** Dysglycemia is associated with poor outcomes; the actual status of dysglycemia of critically ill patients with diabetes should refer to background glycemia. We investigated the effect of difference between mean blood glucose and basic blood glucose upon outcomes.

**Methods:** Glycated hemoglobin A1c (HbA1c) was detected within the first 24 h and converted to A1C-derived average glucose (ADAG) by the equation ADAG = [(HbA1c∗28.7) − 46.7]∗18^−1^; blood glucose measurements were fourth per day during the first 7 days after admission; the mean blood glucose level (Mean), standard deviation (SD), and coefficient of variation (CV) were calculated. GAP were calculated as admission blood glucose and Mean minus ADAG, respectively.

**Results:** Six hundred forty-nine patients were recruited and 428 survived at 28 days; 302 patients with diabetes had greater ADAG, blood glucose at admission (BG_adm_), Mean, SD, CV, GAP, and hypoglycemia incidences. The GAP between Mean and ADAG had superior predictive power, which was decreased in patients with diabetes and increased in patients without diabetes. GAP7 was related to 28-day mortality; the death risk was decreased in patients with diabetes. Patients with lower GAP tended to survive. Nonsurvivors with diabetes suffered higher rate of hypoglycemia than survivors which was the opposite in patients without diabetes.

**Conclusion:** The glycemic GAP between the mean level of blood glucose within the first 7 days in ICU and ADAG was independently associated with 28-day mortality of critically ill patients, which was different between patients with and without diabetes. Hypoglycemia in patients with diabetes should be a concern.

## 1. Introduction

Dysglycemia is a common metabolism disturbance of blood glucose in the intensive care unit (ICU). Studies have shown that hyperglycemia, hypoglycemia, and variability of blood glucose are risk factors for adverse outcomes in critically ill patients [1]. This widely accepted assertion has been doubted in some studies [2], which is more commonly reported in nondiabetes cohort than in diabetes [3]. Marik suggests that hyperglycemia and insulin resistance is an evolutionarily preserved adaptive responsiveness and beneficial for patients striving to survive in the setting of stress [4, 5], so the blood glucose level in ICU is less precise to evaluate clinical status, and variability of blood glucose cannot reflect the association between dysglycemia and mortality in all critically ill individuals. Furthermore, the relationship between hyperglycemia and outcomes was altered by the presence of diabetes [6]; chronic premorbid hyperglycemia increases the risk of hypoglycemia and modifies the association between acute hypoglycemia and mortality [7, 8]; it might be the reason why the correlation between dysglycemia and poor outcome of critically ill patients is more prominent in nondiabetes patients, but it is inconsistent in diabetes patients.

Hyperglycemia in critically ill patients results from acute physiological stress, high baseline blood glucose, or both [9], which makes analysis of the association between blood glucose and outcomes difficult and confused. This prompts us to face the question of what is the most optimal blood glucose target value for critically ill patients. Krinsley, Deane, and Gunst believed that the ideal blood glucose target might depend on pre-existing level of blood glucose control as reflected by glycated hemoglobin A1c (HbA1c) at ICU admission [10]. HbA1c reflects the level of glycemia in the previous 3 months and is a good marker to distinguish background hyperglycemia and normal glycemia [9]. The background hyperglycemia was estimated by HbA1c and defined as A1C-derived average glucose (ADAG) [11]. The glycemic gap, which is calculated by subtracting the ADAG from the value of admission blood glucose, is confirmed to be a good index to depress the impact of chronic hyperglycemia on the disease severity assessment in critically ill patients with diabetes; some researchers reported that elevated glycemic gap can optimally improve the value of the evaluation [12], which helps us to explore the exact relationship between stress-induced hyperglycemia (SIH) and outcomes in critically ill patients with diabetes.

However, the top blood glucose often occurred within the next days after the admission, which means the admission blood glucose could not reflect the actual and extreme severity of SIH. In our two pilot studies which were from the same research project, we found that glycemic gap was independently associated with 28-day mortality of critically ill patients with diabetes, the predictive power extended to 1 year. Also, the predictive power of the glycemic gap was optimized with addition of SOFA (Sequential Organ Failure Assessment) score, which was still confirmed in the diabetes patient cohorts [11, 13]. The objective of the research is to discuss whether GAP_mean_ defined as difference between mean blood glucose level within days after admission to ICU and ADAG is independently associated with mortality or not and how it manifested in diabetes or nondiabetes. Also, the predictive power for mortality of GAP is to be performed.

## 2. Methods

### 2.1. Study Design and Setting

A retrospective observational cohort study of consecutive patients admitted into a 24-bed general ICU in a tertiary teaching hospital in Beijing was conducted between June 1, 2016, and May 31, 2019. The institutional review board for human investigation (Fu Xing Hospital, Capital Medical University IRB, 2015FXHEC-KY012) approved this study and waived the need for informed consent. Protocol was elaborately formulated and closely supervised by the director and performed by all staffs.

### 2.2. Cohort and Data Collection

A population of adults admitted into ICU during a 3-year period of the study, who were estimated to stay over 24 h without oral feeding, were enrolled. Patients were excluded in accordance with the following criteria: (1) pregnant or lactating women, (2) an admission diagnosis of diabetic ketoacidosis or hyperosmolar hyperglycemic syndrome, (3) hyperthyroidism, (4) oral feeding within the first 7 days after admission, (5) corticosteroids administered within 3 months before the admission into ICU, (6) history of ICU stay within 3 months before admission, (7) ICU stay less than 24 h, (8) blood glucose measured less than 3 times in any single day during the first 7 days after admission, (9) HbA1c not obtained, and (10) patients or their representatives signed informed consent of withdrawing life-sustaining treatment within 28 days after the admission.

We recorded the characteristics of enrolled patients including age, sex, body mass index (BMI), APACHE II (Acute Physiology and Chronic Health Evaluation II) score within the first day and the highest SOFA score during the first 7 days (SOFAtop) after admission, diagnoses of diabetes (in accordance with the guideline for the prevention and treatment of Type 2 diabetes mellitus in China 2017), reason for admission, and underlying comorbidities. Laboratory results include arterial blood glucose level during the first 7 days and HbA1c measured within 24 h after admission. Treatment including the way of nutrition support, average daily amount of carbohydrate intake, average daily dosage of insulin (Novolin R), and total dosage of glucocorticoid (converted into dosage of methylprednisolone) for the first 7 days were collected. The primary outcome was whether the patients survived or not on the 28th day after inclusion, and secondary outcomes including duration of ventilator-free days, renal replacement therapy (RRT)–free days, and non-ICU length of stay during 28 days were recorded.

### 2.3. Data Collection of Blood Glucose, HbA1c, and Glycemic Gap

Arterial blood samples were tested by the current method using a blood gas analyzer (GEM PRIMIER3500, Instrumentation Laboratory Co., 180 Hartwell Road, Bedford, Massachusetts 01730-2443, United States) for determining blood glucose level at least every 6 h during the first 7 days and HbA1c (instruments: Tosoh Automated Glycohemoglobin Analyzer HLC-723 G8, 1-37, Fukagawa Minami-machi, Shunan, Yamaguchi, Japan; reagent: Tosoh Hemolyzer, Tosoh Hemoglobin A1c Control Set, 4560, Kaisei-cho, Shunan, Yamaguchi 746-8501, Japan) was detected within the first 24 h. The incidences of hypoglycemia were recorded, including moderate hypoglycemia (MH) which was defined as blood glucose in the range of 2.2–3.3 mmol/L and severe hypoglycemia (SH) with blood glucose lower than 2.2 mmol/L.

The mean blood glucose value within the first 3 days (Mean3) and 5 days (Mean5) and the mean level and the standard deviation (SD) of blood glucose value within 7 days (Mean7 and SD7) were calculated; also, the coefficient of variation of blood glucose during the first 7 days (CV7) was calculated as SD7 divided by Mean7.

HbA1c was converted into ADAG using the equation ADAG = [(HbA1c∗28.7) − 46.7]∗18^−1^. GAP0 was calculated as blood glucose at admission (BG_adm_) minus ADAG as follows: GAP0 = [BG_adm_ − ADAG], GAP3 was Mean3 minus ADAG as GAP3 = [Mean3 − ADAG], GAP5 = [Mean5 − ADAG], and GAP7 = [Mean7 − ADAG].

### 2.4. Statistical Analysis

Data were expressed as mean and SD for normally distributed data and median (interquartile spacing) for nonnormally distributed data and frequencies (percentage) for categorical variables. Analyses were performed by two-tailed Student's *t*-test and chi-square test or Fisher exact test. Receiver operating characteristic (ROC) curves were plotted; the area under the ROC curve (AUC) and 95% confidence interval (CI) were calculated; the DeLong test was performed to analyze the discernibility of the predictive parameters; the threshold of *p* value in multiple comparison was adjusted by Bonferroni correction; Youden's index was applied to ascertain the cutoff value of glycemic gap as an independently predictive factor of 28-day mortality. Logistic regression was used to identify the correlation between the glycemic gap and 28-day mortality; the independent variables with *p* < 0.2 in univariate analysis were brought into the logistic regression analysis as confounding factors for hierarchical correction. Graphs were built using GraphPad Prisma Version 8.0 (GraphPad Software Inc., San Diego, California, United States) and data analyzed using SPSS Statistics Version 24.0 (Chicago, Illinois, United States). A *p* value of < 0.05 was considered statistically significant.

## 3. Results

### 3.1. Study Population and Baseline Characteristics

**Figure 1:**
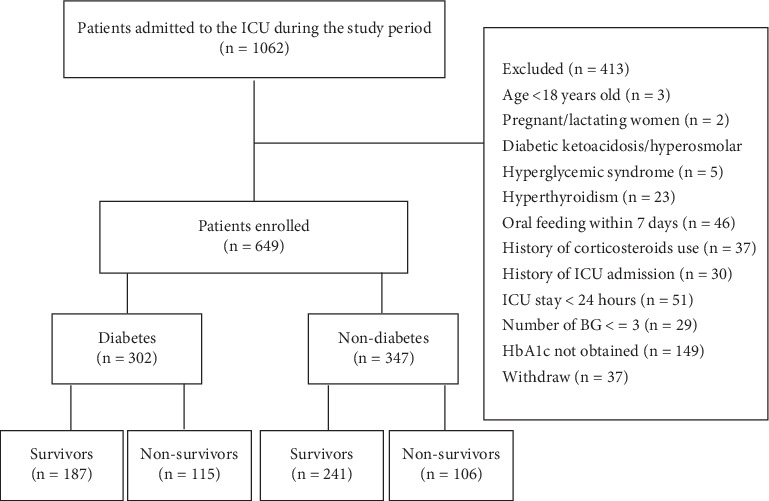
Flowchart of the study.

One thousand and sixty-two patients were admitted to the ICU during the study period; a total of 649 patients were enrolled; 187 (61.9%) of 302 with diabetes and 241 (69.5%) of 347 without diabetes survived on the 28th day; based on which we separated patients into the diabetes and nondiabetes groups and two subgroups survival and nonsurvival, respectively (Figure 1). Blood glucose samples with a number of 20,187 in total and 31.1 per capita were collected; the number of blood glucose sample in patients with diabetes is 9669 in total and 32.0 per capita versus 10,518 in total and 30.3 per capita in nondiabetes (*p* = 0.728).

Patients with diabetes tended to be older and had higher BMI, APACHE II score, SOFAtop scores, proportion with diagnoses of sepsis, comorbidity of cardiac and vascular disease comparing with those without diabetes (Table 1); the characteristics of survivors and nonsurvivors between diabetes and nondiabetes are showed in Table S1.

### 3.2. Data of Blood Glucose

Patients with diabetes had higher blood glucose level, HbA1c, ADAG, and incidence of hypoglycemia than patients without diabetes (Table 2). There were significant differences in HbA1c value and ADAG between the diabetes and nondiabetes groups; higher BG_adm_, Mean3, Mean5, Mean7, and SD7 were found in nonsurvivors as well. Nonsurvivors had equivalent CV7 in diabetes but significantly higher in nondiabetes versus survivors. Besides, incidence of hypoglycemia was higher in survivors with diabetes but oppositely in nondiabetes (Table S2).

### 3.3. Level and Population Distribution of Glycemic Gap

Patients with diabetes had higher GAP at all time points than those without diabetes (Table 2). GAP was higher in nonsurvivors (Table S3) and increased over the first 7 days in both diabetes and nondiabetes groups (Figure S1). The population distribution of patients had similar condition between diabetes and nondiabetes at the point of GAP0; patients with diabetes tended to concentrate in the section of higher GAP3, GAP5, and GAP7 level comparing with the nondiabetes group (Figure S2).

### 3.4. Therapy and Outcome

Patients with diabetes had similar nutrition support ways, carbohydrate intake, and dosage of insulin and glucocorticoid between survivors and nonsurvivors. However, the differences showed up in the nondiabetes group; most survivors were administrated enteral nutrition and received less daily intake of daily dosage of insulin (the protocol of insulin therapy is found in Table S4) and accumulated dosage of glucocorticoid during the first 7 days (Table S5).

There were no significant statistic differences of outcome indicators including the ventilator-free duration, RRT-free duration, and non-ICU stay during 28 days between diabetes and nondiabetes groups in both survivors and nonsurvivors (Table S6).

### 3.5. Predictive Power of Blood Glucose and Glycemic Gap for 28-Day Mortality

**Figure 2:**
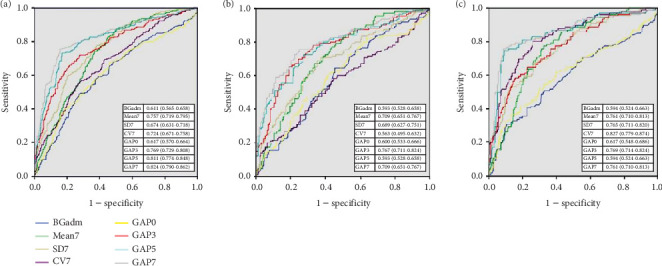
ROC curves of blood glucose and GAP for predicting 28-day mortality. (a) ROC curves for predicting 28-day mortality of all patients. (b) ROC curves for predicting 28-day mortality of the diabetes group. (c) ROC curves for predicting 28-day mortality of the nondiabetes group. GAP value at all time points provided great AUC predictive for 28-day mortality and GAP7 had the maximum.

We plotted ROC curves of BG_adm_, Mean7, SD7, CV7, and GAP for predicting 28-day mortality of all patients (Figure 2(a)), the diabetes group (Figure 2(b)), and the nondiabetes group (Figure 2(c)), among which the AUC of all GAP value at four time points had great predictive power for 28-day mortality and GAP7 had the maximum. The comparison of AUC between GAP7 and others was performed as well; GAP7 provided great predictive power which was similar as GAP5 in both diabetes and nondiabetes groups (Table S7).

The optimal cutoff value of GAP7 to predict 28-day mortality was 3.6 mmol/L (sorted by Youden index with 0.5993), which provided a sensitivity and specificity of 75.6% and 84.3%.

### 3.6. Correlation of GAP7 and 28-Day Mortality

Correlation between GAP7 and mortality was analyzed by multiple logistic regression analysis (Table 3). GAP7 expressed as a continuous variable was positively correlated with 28-day mortality for each 1 mmol/L increase (odds ratio (OR): 1.988, 95% CI: 1.750–2.258); this correlation remained significant adjusted for potential confounders in all patients (OR: 1.595, 95% CI: 1.350–1.885, Model 4); similar trends were shown in both diabetes and nondiabetes and more pronounced in the nondiabetes group. GAP7 was positively correlated with 28-day mortality for each 1 mmol/L increase (OR: 1.645, 95% CI: 1.417–1.909) and OR decreased to 1.424 (95% CI: 1.176–1.726) adjusted for potential confounders in the diabetes group; OR was 2.549 (95% CI: 2.049–3.171) and dropped to 2.315 (95% CI: 1.606–3.336) adjusted for potential confounders in the nondiabetes group.

## 4. Discussion

Patients often experienced abnormal hyperglycemia regardless of whether diagnosed with diabetes or not when they suffered from acute and critical illnesses such as sepsis, multiple trauma, major surgery, acute myocardial infarction (AMI) [14], burns, and stroke [15], whereas SIH is convinced actually related to an increased risk of death especially in patients without diabetes compared with sheer hyperglycemia in patients with diabetes [16, 17]. Researchers believed that critical illness–associated hyperglycemia (CIAH) was associated with increased mortality [18] in patients with “adequately controlled” diabetes; however, elevated blood glucose level itself could not effectively reveal precise correlation between hyperglycemia and mortality in patients with “insufficiently controlled” diabetes [19]. Krinsley suggested that patients with diabetes may benefit from higher blood glucose level than those without diabetes [20] in consideration of increased risk of hypoglycemia [21] and its fatal consequences under inflexible tight blood glucose control strategy [22]. There was no remarkable correlation between a higher level of mean blood glucose within the first 7 days in the ICU and 28-day mortality in our study; the predictive power of mean blood glucose was reasonable but lower than GAP between mean blood glucose and premorbid average blood glucose calculated from HbA1c especially with the presence of diabetes. The conclusion was consistent with results in further study of Krinsley; preadmission glycemia reflected by HbA1c had a significant effect on relationship of ICU glycemia and mortality; different responses to increased mean glycemia supported a personalized approach to blood glucose control practices in ICU [23], even that a target blood glucose value, by itself, may be an inadequate indicator of blood glucose control [24].

Furthermore, nonsurvivors had higher level of blood glucose and needed higher average daily dosage of insulin; however, incidence of hypoglycemia in patients with diabetes was higher than that in those without diabetes, which was consistent with most relevant researches and suggested that restraint of SIH was not rigidly beneficial [4]; patients with diabetes tend to be tolerant of prolonged hyperglycemia and might be adaptive to wider and individualized range of blood glucose [25]; we found that incidence of hypoglycemia was higher in nonsurvivors in the diabetes group, but it was opposite in the nondiabetes group, which inspired the consideration of potential mechanism that patients with diabetes might be more difficult to tolerate MH or SH than patients without diabetes. It is consistent with the conclusions of Stringer that sustained hyperglycemia may potentially increase vulnerability to absolute or even relative hypoglycemia [26]. Hypoglycemia could result in drastic fluctuation of blood glucose and induce more serious cellular impairment [27] and outcomes [28], even was asymptomatic and lasted for a prolonged period [29]; this was convinced associated with ICU mortality, which might explain why subsequent studies failed to replicate the benefit of tight glycemic control in the ICU [30]. Besides, even if hypoglycemia does not occur, fluctuation in blood glucose level is actually present; the correlation of the fluctuation of blood glucose and 28-day mortality and its predictive power for mortality in diabetes and nondiabetes patients with acute and critical illnesses were entirely different. CV7 as the principal indicator of the fluctuation of blood glucose performed significantly correlated with 28-day mortality merely in patients with diabetes, but the correlation was not notable in the nondiabetes cohort. This means that the predictive value of abnormal blood glucose values or related indicators for critically ill patients with or without diabetes and with different levels of blood glucose control is inconsistent; these findings warned researchers to take full account of different responses to standardized glucose control strategies in critically ill patients with chronic hyperglycemia and normoglycemia [23]. The conclusion we obtained inspired us to implement more rational and effective protocols to monitor and control blood glucose to avoid or balance the two extremes which were “uncontrolled hyperglycemia” and “over tightly controlled glucose” [31] for improving outcomes of critically ill patients. The abnormalities of glucose metabolism, such as pre-existing dysglycemia and SIH, were both important factors for outcomes [32]; we therefore need to refer to premorbid condition of blood glucose control to determine optimal target range of blood glucose during early stage of the onset of acute critically ill patients. This was the reason that we had to search for other variables to detect the correlation between blood glucose and mortality, especially for different predictive power in diabetic and nondiabetic patients.

Some researchers believed that admission blood glucose was associated with increased mortality of critically ill patients [33], however, our study did not witness a coincident conclusion. Hyperglycemia was the result of premorbid hyperglycemia and stress response [34, 35], since the absolute value of blood glucose was not independently associated with short or long term mortality especially in diabetic patients, the quality of blood glucose control was elementary for analyzing the correlation between blood glucose level and mortality after the onset of acute critical diseases. HbA1c as an indicator that represents premorbid chronic hyperglycemia is not affected by stress or fasting status in a short term, which is relatively stable within a day and day-to-day variations [36, 37]. HbA1c therefore deserves to be a crucial index to recognize chronic hyperglycemia and stress hyperglycemia [38] and routinely performed to calculate ADAG. The difference between admission blood glucose and ADAG was confirmed to be associated with adverse outcomes [39, 40], by which the contradiction between our conclusion and those of others might be explained. The state of chronic glycemia in critically ill patients provides important clinical information on severity of critical illness–associated dysglycemia [22]. Nonetheless, we found pronounced correlation between glycemic GAP during early stage in ICU and 28-day mortality but GAP0—difference between admission blood glucose and ADAG—was not the best in all glycemic GAP at four time points. Moreover, all glycemic GAP between mean level of blood glucose in 3, 5, and 7 days except admission blood glucose and ADAG had great predictive power for 28-day mortality especially in patients without diabetes comparing with the diabetic cohort. This might be derived from diverse reactivity, severity, and progression of critical illnesses in patients with different basic health conditions; a single point of blood glucose value could not reflect the reality and variation veritably and timely with numerous impacted factors and unforeseen circumstances; particularly, the first blood glucose value might be affected by some factors, such as the timing of admission into the ICU and treatment to control blood glucose before the onset of the attack; besides, attention should be focused on subhealth status and vital organ function before aggravation. The mean level of blood glucose during the first several days in the ICU combined with ADAG could be more practical for analyzing the real association between dysglycemia and mortality in the ICU, whereas researchers could acquire more comprehensive information of the patients effectively than the first single blood glucose sample.

### 4.1. Limitations

First, selection bias may exist in this single-center study with a limited number of samples. Second, there remains lack of consensus on the target range of blood glucose. Insulin was administered through intravenous way continuously or subcutaneous way intermittently to achieve target blood glucose that ranged from 8.0 to 10.0 mmol/L in our study, which was considered as the safe range for critically ill patients; however, some disagreed with this target level. Additionally, the percentage of time reaching the blood glucose target range was not analyzed as well. Third, some medications that may affect blood glucose were not analyzed, such as catecholamines, diuretic, analgesics, and antibiotics. Fourth, the proportion of enteral and parenteral carbohydrate intake was not analyzed. It is necessary to carry out multicenter studies to increase the sample size and obtain more clinical data to demonstrate the relationship between dysglycemia and adverse outcomes and improve the strategy of monitoring and controlling blood glucose of critically ill patients with or without diabetes in further researches; subgroup analysis of effects of relative medications or other treatments may be needed as well.

## 5. Conclusions

In this study, an elevated glycemic gap between mean blood glucose level in the first 7 days after admission to the ICU and ADAG was independently associated with 28-day mortality in critically ill patients, especially in those without diabetes; the predictive power for mortality was superior to other known glycemic indicators associated with mortality including GAP_adm_ (difference between admission blood glucose and ADAG) regardless of whether with diabetes or not. Furthermore, incidence of hypoglycemia was higher in nonsurvivors with diabetes but reversed in patients without diabetes; it reminded us to take more attention to serious harm and warning of hypoglycemia in critically ill patients with diabetes.

## Figures and Tables

**Table 1:** Baseline characteristics of the patients enrolled.

**Baseline characteristics**	**Diabetes (** **n** = 302**)**	**Nondiabetes (** **n** = 347**)**	**All patients (** **n** = 649**)**	**p** ** value**
Sex (male), *n* (%)	172 (57.0)	214 (61.7)	386 (59.5)	0.155
Age (y)	81.0 (71.8, 85.3)	74.0 (58.0, 82)	79.0 (67.0, 85.0)	*< 0.001*⁣^∗^
BMI (kg/m^2^)	24.2 (21.6, 26.1)	23.2 ± 3.9	24.2 (21.5, 26.0)	*0.027*⁣^∗^
APACHE II score	21.0 (15.0, 27.0)	17.0 (12.0, 24.0)	20.0 (15.0, 26.0)	*< 0.001*⁣^∗^
SOFAtop score	10.0 (6.0, 12.0)	6.0 (3.0, 9.0)	8.0 (5.0, 12.0)	*< 0.001*⁣^∗^
Surgical patients, *n* (%)	34 (11.3)	45 (13.0)	79 (12.2)	0.551
Reason for ICU admission, *n* (%)				
Sepsis	84 (27.8)	63 (18.2)	147 (22.7)	*0.004*⁣^∗^
Thoracic or respiratory disease	80 (26.5)	115 (33.1)	195 (30.0)	0.125
Cardiac and vascular disease	63 (20.9)	69 (19.9)	132 (20.3)	0.096
Neurologic disease	22 (7.3)	32 (9.2)	54 (8.3)	0.646
Renal dysfunction	18 (6.0)	16 (4.6)	34 (5.2)	0.483
Gastrointestinal disease	17 (5.6)	23 (6.6)	40 (6.2)	0.881
Postoperative care	10 (3.3)	9 (2.6)	19 (2.9)	0.645
Other	8 (2.6)	20 (5.8)	28 (4.3)	0.369
Comorbidities, *n* (%)				
Respiratory disease	66 (21.9)	66 (19.0)	132 (20.3)	0.381
Cardiac and vascular disease	269 (89.1)	239 (68.9)	508 (78.3)	*< 0.001*⁣^∗^
Cerebrovascular disease	190 (62.9)	160 (46.1)	350 (53.9)	0.257
Chronic renal disease	113 (37.4)	108 (31.1)	221 (34.1)	0.097
Gastrointestinal disease	21 (7.0)	26 (7.5)	47 (7.2)	0.880
Malignancy	61 (20.2)	65 (18.7)	126 (19.4)	0.691

*Note:* APACHE II score: Acute Physiology and Chronic Health Evaluation II score, SOFAtop score: the top level of Sequential Organ Failure Assessment score.

⁣^∗^*p* < 0.05.

**Table 2:** Data of blood glucose levels, HbA1c, ADAG, and GAP of the patients enrolled.

**Blood glucose variables**	**Diabetes (** **n** = 302**)**	**Nondiabetes (** **n** = 347**)**	**All patients (** **n** = 649**)**	**p** ** value**
BG_adm_ (mmol/L)	11.8 (8.9, 15.4)	8.0 (6.4, 10.6)	9.5 (7.0, 13.0)	*< 0.001*⁣^∗^
Mean3 (mmol/L)	12.1 ± 3.1	8.7 (7.3, 11.1)	10.0 (8.1, 12.8)	*< 0.001*⁣^∗^
Mean5 (mmol/L)	12.1 (9.9, 13.9)	8.8 (7.3, 11.3)	10.4 (7.9, 12.7)	*< 0.001*⁣^∗^
Mean7 (mmol/L)	12.3 (10.2, 13.9)	8.9 (7.3, 11.2)	10.6 (8.0, 12.8)	*< 0.001*⁣^∗^
SD7 (mmol/L)	3.4 (2.6, 4.6)	2.0 (1.5, 3.1)	2.7 (1.8, 3.9)	*< 0.001*⁣^∗^
CV7 (%)	30.1 (23.2, 36.4)	23.3 (18.4, 29.9)	25.7 (20.0, 33.0)	*< 0.001*⁣^∗^
HbA1c (%)	7.3 (6.4, 8.2)	6.0 (5.4, 6.7)	6.5 (5.7, 7.5)	*< 0.001*⁣^∗^
ADAG (mmol/L)	9.0 (7.6, 10.5)	7.0 (6.0, 8.1)	7.8 (6.5, 9.4)	*< 0.001*⁣^∗^
MH/SH, *n* (%)	63 (20.9)	46 (13.3)	109 (16.8)	*0.007*⁣^∗^
GAP0 (mmol/L)	2.7 (−0.2, 6.0)	1.4 (−0.1, 3.2)	1.8 (−0.1, 4.5)	*0.001*⁣^∗^
GAP3 (mmol/L)	3.1 (1.2, 4.4)	2.1 (0.9, 3.6)	2.5 ± 2.3	*< 0.001*⁣^∗^
GAP5 (mmol/L)	3.2 (1.5, 4.2)	2.3 ± 1.9	2.7 (1.1, 4.0)	*< 0.001*⁣^∗^
GAP7 (mmol/L)	3.3 (0.8, 4.2)	2.3 (0.9, 3.7)	2.8 (0.9, 3.9)	*0.006*⁣^∗^

*Note:* BG_adm_: blood glucose at admission into the ICU, Mean3: mean glucose level within the first 3 days in the ICU, Mean5: mean glucose level within the first 5 days in the ICU, Mean7: mean glucose level within the first 7 days in the ICU, SD7: standard deviation of blood glucose within the first 7 days in the ICU, CV7: variation coefficient of blood glucose within the first 7 days in the ICU (SD7/Mean7), MH: moderate hypoglycemia, blood glucose: 2.2–3.3 mmol/L, SH: severe hypoglycemia, blood glucose: < 2.2 mmol/L, GAP0: glycemic gap between BG at admission and ADAG, GAP3: glycemic gap between Mean3 and ADAG, GAP5: glycemic gap between Mean5 and ADAG, GAP7: glycemic gap between Mean7 and ADAG.

Abbreviation: ADAG, A1C-derived average glucose.

⁣^∗^*p* < 0.05.

**Table 3:** Multiple logistic regression analysis of the correlation between GAP7 and 28-day mortality.

**Model**	**All patients**		**Diabetes**		**Nondiabetes**	
	**OR (95% CI)**	**p** ** value**	**OR (95% CI)**	**p** ** value**	**OR (95% CI)**	**p** ** value**
Model 1	1.988 (1.750–2.258)	*< 0.001*⁣^∗^	1.645 (1.417–1.909)	*< 0.001*⁣^∗^	2.549 (2.049–3.171)	*< 0.001*⁣^∗^
Model 2	1.874 (1.634–2.150)	*< 0.001*⁣^∗^	1.617 (1.393–1.877)	*< 0.001*⁣^∗^	2.634 (1.952–3.553)	*< 0.001*⁣^∗^
Model 3	1.643 (1.402–1.926)	*< 0.001*⁣^∗^	1.453 (1.207–1.748)	*< 0.001*⁣^∗^	2.286 (1.635–3.196)	*< 0.001*⁣^∗^
Model 4	1.595 (1.350–1.885)	*< 0.001*⁣^∗^	1.424 (1.176–1.726)	*< 0.001*⁣^∗^	2.315 (1.606–3.336)	*< 0.001*⁣^∗^

*Note:* Mean7: mean glucose level within the first 7 days in the ICU, GAP7: glycemic gap between Mean7 and ADAG. Model 1: GAP7 was entered as a continuous variable into analysis, no adjustment. Model 2: adjusted as for Model 1, additionally adjusted for age and body mass index (BMI). Model 3: adjusted as for Model 2, additionally adjusted for Acute Physiology and Chronic Health Evaluation II score (APACHE II score), the top level of Sequential Organ Failure Assessment score (SOFAtop score), surgical, comorbidities (cardiac and vascular disease vs. without cardiac and vascular disease), and reason of admission (sepsis vs. nonsepsis). Model 4: adjusted as for Model 3, additionally adjusted for nutrition support ways, average daily amount of carbohydrate intake, average daily dosage of insulin (Novolin R), and total dosage of glucocorticoid (converted into dosage of methylprednisolone).

Abbreviations: ADAG, A1C-derived average glucose; CI, confidence interval; OR, odds ratio.

⁣^∗^*p* < 0.05.

## Data Availability

The data used to support the findings of this study are available from the corresponding author upon request.

## Nomenclature

ADAG: A1c-derived average glucose
APACHE II: Acute Physiology and Chronic Health Evaluation II
AUC: area under the ROC curve
BG_adm_: blood glucose at admission
BMI: body mass index
CI: confidence interval
CV: coefficient of variation
GAP_adm_: GAP between BG_adm_ and ADAG
GAP_mean_: GAP between MGL and ADAG
HbA1c: glycated hemoglobin A1c
ICU: intensive care unit
MH: moderate hypoglycemia
MODS: multiple organ dysfunction syndrome
ROC curve: receiver operating characteristic curve
RRT: renal replacement therapy
SD: standard deviation
SIH: stress-induced hyperglycemia
SH: severe hypoglycemia
SOFA: Sequential Organ Failure Assessment
