# Supplementary material for: The Value of Glycemic Gap for Predicting Mortality in ICU in Patients With and Without Diabetes
Source: J Diabetes Res. 2025 Feb 14;2025:4563928. doi: 10.1155/jdr/4563928 (PMC11845263; doi:10.1155/jdr/4563928)
Supplement: Supporting Information — Additional supporting information can be found online in the Supporting Information section. [file 4563928.f1.doc]

**Supplementary Appendix**

This appendix was provided by all authors to give readers additional information about our work.

Supplement to: Ran Lou, Li Jiang, Meiping Wang, et al. Glycemic Gap in ICU-similar and different in patients with and without Diabetes.

**The value of Glycemic Gap for predicting mortality in ICU in patients with and without Diabetes**

**SUPPLEMENTARY MATERIAL**

Ran Lou^1^

Li Jiang^1^,*

Meiping Wang^2^

Tingting Wang^1^

Quan Si^1^

Weixue Su^1^

Nan Wang^1^

Yuyan Liu^1^

Ting Chen^1^

Qi Jiang^3^

Bo Zhu^3^

^1^Department of Crtical Care Medicine, Xuanwu Hospital Capital Medical University. 45Changchun Street, Xicheng District, Beijing 100053, China;

^2^ Department of Epidemiology and Health Statistics, School of Public Health, Capital Medical University. 10 Xitoutiao, Youanmenwai, Fengtai District, Beijing 100069, China;

^3^Department of Critical Care Medicine, Fu Xing Hospital, Capital Medical University. 20A Fuxingmenwai Street, Xicheng District, Beijing 100038, China.

**SUPPLEMENTARY MATERIAL**

**This supple**m**ent file has additional information on results, which shows the information that cannot be fully displayed in the main manuscript due to the limited space. The information listed in this section mainly includes the characteristics of the patients enrolled, data of some blood glucose related indicators, and therapy for the control of the blood glucose and the support of organs function. This section is organized as:**

**I)** **DESCRIPTION OF THE STUDIED POPULATION: (***Tables S1***)**

**II) ADDITIONAL RESULTS / ANALYSIS REFERRED TO IN THE MAIN MANUSCRIPT:**

1. **Data of Blood Glucose Levels, HbA1c, and ADAG of the Patients Enrolled** (*Tables S2*)
2. **Level and population distribution of glycemic gap** (*Tables S3, Figure S1-S2*)
3. **The protocol of insulin therapy** *(Table S4)*
4. **Therapy of Patients Enrolled** (*Tables S5*)
5. **Outcome of the Survivors and Non-survivors with and without Diabetes** (*Tables S7*)
6. **The Predictors for 28-day Mortality and Comparison of AUC of the Predictors** (*Tables S6*)

**Table S1: Baseline Characteristics of the Survivors and Non-survivors with and without Diabetes**

The diabetes group had similar tendency in age and proportion of patients with major diagnose of sepsis comparing with non-diabetes group in survivors but not in non-survivors, meanwhile, a higher proportion of comorbidity of cardiac and vascular disease and cerebrovascular disease was seen in patients with diabetes in both survivors and non-survivors .

| Baseline Characteristics | Survivors | | | |  | Non-survivors | | | |
| --- | --- | --- | --- | --- | --- | --- | --- | --- | --- |
|  | Diabetes  (n=187) | Non-diabetes  (n=241) | All suvivors  (n=428) | *P*-value |  | Diabetes  (n=115) | Non-diabetes  (n=106) | All non-suvivors  (n=221) | *P*-value |
| Sex (male), n (%) | 110(58.8) | 148(61.4) | 258(60.3) | 0.478 |  | 62(53.9) | 66(62.3) | 128(57.9) | 0.058 |
| Age (y) | 79.0(68.0,85.0) | 71.0(55.0,81.0) | 77.0(64.0,84.0) | *0.008** |  | 83.0(77.0,86.8) | 78.0(67.0,83.5) | 82.0(76.0,86.0) | 0.255 |
| BMI (Kg/m^2^) | 24.2(21.7,26.1) | 23.1(20.8,25.8) | 23.7(21.5,26.1) | 0.272 |  | 23.9(21.5,26.1) | 20.9±3.7 | 23.8(21.5,26.1) | 0.624 |
| APACHE II score | 19.0(14.0,24.0) | 16.0(10.0,21.0) | 18.0(13.0,23.0) | 0.061 |  | 25.4±8.7 | 26.0±8.6 | 25.7±8.6 | 0.800 |
| SOFAtop score | 7.0(5.0,10.0) | 5.0(3.0,7.3) | 6.0(4.0,9.0) | 0.112 |  | 12.0(10.0,15.0) | 9.7±4.9 | 12.0(9.8,15.0) | 0.074 |
| Surgical patients, n (%) | 24(12.8) | 30(12.4) | 54(12.6) | 0.671 |  | 10(8.7) | 15(14.2) | 25(11.3) | 0.368 |
| Reason for ICU admission, n (%) |  |  |  |  |  |  |  |  |  |
| Sepsis | 43(23.0) | 29(12.0) | 72(16.8) | *0.003** |  | 41(35.7) | 34(32.1) | 75(33.9) | 0.480 |
| Thoracic or respiratory disease | 56(29.9) | 83(34.4) | 139(32.5) | 0.350 |  | 24(20.9) | 32(30.2) | 56(25.3) | 0.289 |
| Cardiac and vascular disease | 32(17.1) | 44(18.3) | 76(17.8) | 0.876 |  | 31(27.0) | 25(23.6) | 56(25.3) | 0.322 |
| Neurologic disease | 12(6.4) | 21(8.7) | 33(7.7) | 0.515 |  | 10(8.7) | 11(10.4) | 21(9.5) | 0.694 |
| Renal dysfunction | 14(7.5) | 10(4.1) | 24(5.6) | 0.245 |  | 4(3.5) | 6(5.7) | 10(4.5) | 0.526 |
| Gastrointestinal disease | 14(7.5) | 13(5.4) | 27(6.3) | 0.576 |  | 3(2.6) | 10(9.4) | 13(5.9) | 0.113 |
| Postoperatie care | 9(4.8) | 7(2.9) | 16(3.7) | 0.317 |  | 1(0.9) | 2(1.9) | 3(1.4) | 0.608 |
| Other | 7(3.7) | 11(4.6) | 18(4.2) | 0.688 |  | 1(0.9) | 9(8.5) | 10(4.5) | 0.097 |
| Comorbidities, n (%) |  |  |  |  |  |  |  |  |  |
| Respiratory disease | 39(20.9) | 41(17.0) | 80(18.7) | 0.320 |  | 27(23.5) | 25(23.4) | 52(23.5) | 0.985 |
| Cardiac and vascular disease | 162(86.6) | 160(66.4) | 322(75.2) | *＜0.001** |  | 107(93.0) | 79(74.5) | 186(84.2) | *＜0.001** |
| Cerebrovascular disease | 119(63.6) | 113(46.9) | 232(54.2) | *0.001** |  | 71(61.7) | 47(44.3) | 118(53.4) | *0.011** |
| Chronic renal disease | 65(34.8) | 66(27.4) | 131(30.6) | 0.113 |  | 48(41.7) | 42(39.6) | 90(40.7) | 0.785 |
| Gastrointestinal disease | 14(7.5) | 17(7.1) | 30(7.0) | 0.707 |  | 7(6.1) | 9(8.5) | 16(7.2) | 0.678 |
| Malignancy | 39(20.9) | 43(17.8) | 82(19.2) | 0.459 |  | 22(19.1) | 22(20.8) | 44(19.9) | 0.866 |

APACHE II score: Acute Physiology and Chronic Health Evaluation II score, SOFAtop score: the top level of Sequential Organ Failure Assessment score

**P* < 0.05

**Table S2. Data of Blood Glucose Levels, HbA1c, and ADAG of the Survivors and Non-survivors with and without diabetes**

There were no significant differences in HbA1c value and ADAG between the diabetes and non-diabetes group, higher levels of BGadm, Mean3, Mean5, Mean7, and SD7 were found in non-survivors in both diabetes and non-diabetes group. Nevertheless, the performances of CV7 and the incidence of hypoglycemia were different among diabetes and non-diabetes group, patients with diabetes had similar level of CV7 between survivors and non-survivors, but non-survivors without diabetes had significantly higher CV7 than survivors. Besides, the incidence of hypoglycemia was higher in survivors with diabetes but oppositely in non-diabetes.

| Blood Glucose Variables | Diabetes | | | |  | Non-diabetes | | | |
| --- | --- | --- | --- | --- | --- | --- | --- | --- | --- |
|  | Survivors  (n=187) | Non-survivors  (n=115) | All diabetes  (n=302) | *P*-value |  | Survivors  (n=241) | Non-survivors  (n=106) | All non- diabetes  (n=347) | *P*-value |
| BGadm (mmol/L) | 11.0(8.5, 15.0) | 12.7(10.1, 16.3) | 11.8(8.9,15.4) | *0.007** |  | 7.8(6.3,10.0) | 9.1(6.9,12.8) | 8.0(6.4,10.6) | *0.005** |
| Mean3 (mmol/L) | 10.8±3.0 | 12.8±2.8 | 12.1±3.1 | *＜0.001** |  | 7.8(6.7,9.3) | 9.4(7.9,12.4) | 8.7(7.3,11.1) | *0.001** |
| Mean5 (mmol/L) | 10.5±3.1 | 12.8±2.4 | 12.1(9.9,13.9) | *＜0.001** |  | 7.7(6.8,9.1) | 9.5±2.1 | 8.8(7.3,11.3) | *＜0.001** |
| Mean7 (mmol/L) | 11.2±2.9 | 13.3±2.5 | 12.3(10.2,13.9) | *＜0.001** |  | 7.6(6.7,9.1) | 10.9±2.3 | 8.9(7.3,11.2) | *＜0.001** |
| SD7 (mmol/L) | 3.1(2.5,4.2) | 4.2±1.6 | 3.4(2.6, 4.6) | *＜0.001** |  | 1.8(1.3,2.3) | 3.4±1.3 | 2.0(1.5,3.1) | *＜0.001** |
| CV7 (%) | 29.4±0.1 | 32.1±0.1 | 30.1(23.2, 36.4) | 0.066 |  | 20.8(16.6,26.6) | 30.7±0.1 | 23.3(18.4,29.9) | *＜0.001** |
| HbA1c (%) | 7.2(6.3,8.3) | 7.4(6.6,8.1) | 7.3(6.4,8.2) | 0.487 |  | 5.9(5.4,6.7) | 6.1±0.9 | 6.0(5.4,6.7) | 0.625 |
| ADAG (mmol/L) | 8.9(7.5, 10.6) | 9.0(7.9,10.3) | 9.0(7.6,10.5) | 0.512 |  | 6.8(6.0,8.1) | 7.1±1.5 | 7.0(6.0,8.1) | 0.625 |
| MH/SH, n(%) | 16(8.6) | 47(40.9) | 63(20.9) | *＜0.001** |  | 37(15.4) | 9(8.5) | 46(13.3) | *0.01** |

BGadm: blood glucose at admission into ICU, Mean3: mean glucose level within first 3 days in ICU, Mean5: mean glucose level within first 5 days in ICU, Mean7: mean glucose level within first 7 days in ICU, SD7: standard deviation of blood glucose within first 7 days in ICU, CV7: variation coefficient of blood glucose within first 7 days in ICU (SD7/Mean7), ADAG: A1C-derived average glucose, MH: moderate hypoglycemia, blood glucose:2.2-3.3mmol/L, SH: severe hypoglycemia, blood glucose:<2.2mmol/L

**P* < 0.05

**Level and population distribution of glycemic gap**

Non-survivors had higher level of GAP in both diabetes and non-diabetes group than survivors (*Table S3*).The level of GAP increased over the period of the first 7 days in ICU in both diabetes and non-diabetes group (*Figure S1*).

**Table S3. Data of Glycemic GAP of the Survivors and Non-survivors with and without diabetes**

| Glycemic Gap | Diabetes | | | |  | Non-diabetes | | | |
| --- | --- | --- | --- | --- | --- | --- | --- | --- | --- |
|  | Survivors  (n=187) | Non-survivors  (n=115) | All diabetes  (n=302) | *P*-value |  | Survivors  (n=241) | Non-survivors  (n=106) | All non- diabetes  (n=347) | *P*-value |
| GAP0 (mmol/L) | 2.0(-0.7,5.2) | 3.3(0.9,6.3) | 2.7(-0.2, 6.0) | *0.004** |  | 0.8(-0.1,2.3) | 1.9(0.3,4.0) | 1.4(-0.1,3.2) | *＜0.001** |
| GAP3 (mmol/L) | 2.2(0.6,3.6) | 4.2(3.6,5.0) | 3.1(1.2,4.4) | *＜0.001** |  | 1.5(0.6,2.9) | 2.8±1.5 | 2.1(0.9,3.6) | *＜0.001** |
| GAP5 (mmol/L) | 2.4(0.5, 3.5) | 4.2(3.7,5.0) | 3.2(1.5,4.2) | *＜0.001** |  | 1.6±1.4 | 3.5(2.2,4.1) | 2.3±1.9 | *＜0.001** |
| GAP7 (mmol/L) | 2.5(0.7,3.5) | 4.3(3.5,5.4) | 3.3(0.8,4.2) | *＜0.001** |  | 1.6±1.5 | 3.7(2.3,4.1) | 2.3(0.9,3.7) | *＜0.001** |

GAP0: glycemic gap between BG at admission and ADAG, GAP3: glycemic gap between Mean3 and ADAG, GAP5: glycemic gap between Mean5 and ADAG, GAP7: glycemic gap between Mean7 and ADAG

*P < 0.05


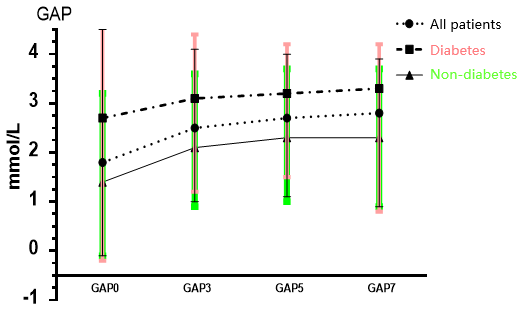


**Figure S1. The Upward Trend of Glycemic GAP**

GAP0: glycemic gap between BG at admission and ADAG, GAP3: glycemic gap between Mean3 and ADAG, GAP5: glycemic gap between Mean5 and ADAG, GAP7: glycemic gap between Mean7 and ADAG


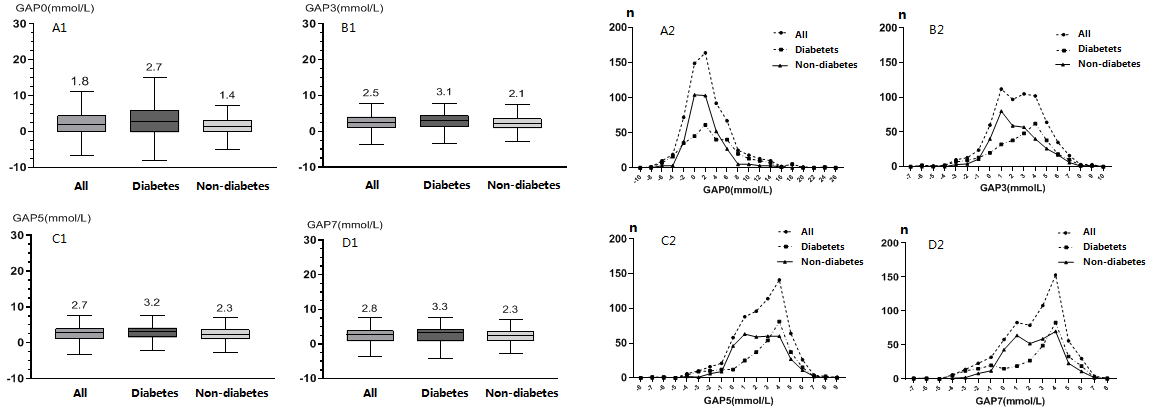


**Figure S2. The Level of Glycemic GAP of Patients and Population Distribution at Four Time Points in Diabetes and Non-diabetes Group**

A1: Glycemic GAP at admitted into ICU, B1: Glycemic GAP in first 3 days in ICU, C1: Glycemic GAP in first 5 days in ICU, D1: Glycemic GAP in first 7 days in ICU

A2: Population Distribution at admitted into ICU, B2: Population Distribution in first 3 days in ICU, C2: Population Distribution in first 5 days in ICU, D2: Population Distribution in First 7 days in ICU

**Table S4. Therapy of Patients Enrolled**

| **Blood Glucose (mmol/L)** | **Low Dose** | **Moderate Dose** | **High Dose** |
| --- | --- | --- | --- |
| 3.3 - 6.1 | No insulin | No insulin | No insulin |
| 6.2 - 8.3 | 2 units | 4 units | 6 units |
| 8.4 - 11.1 | 4 units | 8 units | 10 units |
| 11.2 - 13.8 | 6 units | 10 units | 12 units |
| 13.9 - 16.6 | 8 units | 12 units | 15 units |
| 16.7 - 19.4 | 10 units | 14 units | 18 units |
| ＞ 19.4 | 12 units | 16 units | 20 units |

**Table S5. Therapy of Patients Enrolled**

Patients with diabetes had no difference of nutrition support ways, carbohydrate intake, dosage of insulin and glucocorticoid between survivors and non-survivors. However, the differences showed up in the non-diabetes group, most survivors were administrated enteral nutrition and received less daily intake of daily dosage of insulin (Novolin R) and accumulated dosage of glucocorticoid (converted into the dosage of Methylprednisolone) during the first 7 days in ICU than non-survivors which was the same as patients with diabetes (*Table S5*).

| Therapy | Diabetes | | | |  | Non-diabetes | | | |
| --- | --- | --- | --- | --- | --- | --- | --- | --- | --- |
|  | Survivors  (n=187) | Non-survivors  (n=115) | All diabetes  (n=302) | *P*-value |  | Survivors  (n=241) | Non-survivors  (n=106) | All non- diabetes  (n=347) | *P*-value |
| Nutrition support |  |  |  |  |  |  |  |  |  |
| None, n(%) | 9(4.8) | 12(10.4) | 21(7.0) | 0.101 |  | 9(3.7) | 14(13.2) | 23(6.6) | *0.002** |
| Enteral nutrition, n(%) | 147(78.6) | 86(74.8) | 233(77.2) | 0.481 |  | 195(80.9) | 72(67.9) | 267(76.9) | *0.012** |
| Parenteral nutrition, n(%) | 10(5.3) | 9(7.8) | 19(6.3) | 0.466 |  | 6(2.5) | 10(9.4) | 16(4.6) | *0.009** |
| Enteral and parenteral nutrition, n(%) | 21(11.2) | 8(7.0) | 29(9.6) | 0.314 |  | 31(12.9) | 10(9.4) | 41(11.8) | 0.470 |
| Carbohydrate intake (Kcal/kg) | 2.3(2.0,2.7) | 2.2±0.5 | 2.3(2.0,2.6) | 0.391 |  | 2.3(1.9,2.5) | 2.3±0.5 | 2.3(1.9,2.6) | 0.479 |
| Insulin daily dosage (u) | 16.0(0,39.2) | 26.7(4.0,40.0) | 20.0(1.1,40.0) | *0.031** |  | 0(0,7.0) | 9.5(0,23.2) | 0(0,12.5) | *＜0.001** |
| Glucocorticoid dosage (mg) | 0(0,66.7) | 40.0(0,160.0) | 26.7(0,110.0) | *0.001** |  | 0(0,80.0) | 63.3(0,240.0) | 0(0,140.0) | *＜0.001** |

*P < 0.05

**Table S6. Outcome of the Survivors and Non-survivors with and without Diabetes**

There were no significant statistic differences of outcome indicators including the duration of ventilator-free, renal replacement therapy (RRT)-free, and non-ICU stay during 28 days between diabetes and non-diabetes groups in both survivors and non-survivors (*Table S6*).

| Outcome | Survivors | | | |  | Non-survivors | | | |
| --- | --- | --- | --- | --- | --- | --- | --- | --- | --- |
|  | Diabetes  (n=187) | Non-diabetes  (n=241) | All survivors  (n=428) | *P*-value |  | Diabetes  (n=115) | Non-diabetes  (n=106) | All non-survivors  (n=221) | *P*-value |
| Duration of ventilator-free (h) | 558.0(368.0, 657.0) | 579.0(356.0,672.0) | 571.5(361.0,671.8) | 0.143 |  | 0(1.0,38.0) | 0(0,36.8) | 1.0(0,37.0) | 0.635 |
| Duration of RRT-free (h) | 672.0(672.0,672.0) | 672.0(672.0,672.0) | 672.0(672.0,672.0) | 0.395 |  | 672.0(637.0,672.0) | 672.0(610.8,672.0) | 672.0(629.0,672.0) | 0.457 |
| ICU-free days (d) | 16.0(2.0, 21.0) | 16.0(3.5,21.0) | 16.0(2.0,21.0) | 0.545 |  | 0(0,0) | 0(0,0) | 0(0,0) | 0.219 |

RRT: renal replacement therapy

**Table S7. The Predictors for 28-day Mortality and Comparison of AUC of the Predictors**

We plotted the ROC curves of BGadm, mean7, SD7, CV7, and GAP for predicting the 28-day mortality of all patients enrolled, among which the AUC of all of the GAP value at four time points had great predictive power for 28-day mortality and GAP7 had the maximum, the comparison of AUC between GAP7 and others was performed as well, GAP7 was the best predictive power which was similar as GAP5 in both diabetes and non-diabetes group (*Table S7*).

| Blood Glucose Variables | All Patients | | |  | Diabetes | | |  | Non-diabetes | | |
| --- | --- | --- | --- | --- | --- | --- | --- | --- | --- | --- | --- |
|  | OR (95%CI) | Difference of AUC^#^ | *P*-value^#^ |  | OR (95%CI) | Difference of AUC^#^ | *P*-value^#^ |  | OR (95%CI) | Difference of AUC^#^ | *P*-value^#^ |
| BGadm | 0.611(0.565-0.658) | 0.213(0.173–0.261) | *＜0.001*** |  | 0.593(0.528-0.658) | 0.206(0.167–0.253) | *＜0.001*** |  | 0.594(0.524-0.663) | 0.263(0.198–0.297) | *＜0.001*** |
| Mean7 | 0.757(0.719-0.795) | 0.067(0.059-0.134) | *＜0.001*** |  | 0.709(0.651-0.767) | 0.099(0.020-0.143) | *＜0.001*** |  | 0.761(0.710-0.813) | 0.096(0.099-0.167) | *＜0.001*** |
| SD7 | 0.674(0.631–0.718) | 0.150(0.014–0.201) | *＜0.001*** |  | 0.689(0.627–0.751) | 0.109(0.011–0.182) | *＜0.001*** |  | 0.765(0.711–0.820) | 0.092(0.012–0.186) | *＜0.001*** |
| CV7 | 0.724(0.671-0.758) | 0.107(0.008-0.194) | *＜0.001*** |  | 0.563(0.495-0.632) | 0.236(0.135-0.224) | *＜0.001*** |  | 0.827(0.779-0.874) | 0.030(0.002-0.153) | 0.030 |
| GAP0 | 0.617(0.570-0.664) | 0.207(0.161-0.257) | *＜0.001*** |  | 0.600(0.533-0.666) | 0.199(0.127-0.194) | *＜0.001*** |  | 0.617(0.548-0.686) | 0.240(0.121-0.203) | *＜0.001*** |
| GAP3 | 0.769(0.729-0.808) | 0.055(0.033-0.081) | *＜0.001*** |  | 0.767(0.711-0.824) | 0.032(0.029-0.103) | 0.021 |  | 0.769(0.714-0.824) | 0.088(0.048-0.103) | *＜0.001*** |
| GAP5 | 0.811(0.774-0.848) | 0.013(-0.003-0.033) | 0.011 |  | 0.772(0.717-0.828) | 0.027(0.031-0.077) | 0.020 |  | 0.851(0.804-0.899) | 0.001(-0.004-0.033) | 0.251 |
| GAP7 | 0.824(0.790-0.862) | *-* | *-* |  | 0.799(0.746-0.853) | *-* | *-* |  | 0.857(0.809-0.905) | *-* | *-* |

BGadm: blood glucose at admission into ICU, Mean3: mean glucose level within first 3 days in ICU, Mean5: mean glucose level within first 5 days in ICU, Mean7: mean glucose level within first 7 days in ICU, SD7: standard deviation of blood glucose within first 7 days in ICU, CV7: variation coefficient of blood glucose within first 7 days in ICU (SD7/Mean7), ADAG: A1C-derived average glucose, MH: moderate hypoglycemia, blood glucose:2.2-3.3mmol/L, SH: severe hypoglycemia, blood glucose:<2.2mmol/L

#AUC compared with that of GAP7

***P* < 0.008, adjusted by Bonferroni correction
